# Potential Mechanism Underlying Exercise Upregulated Circulating Blood Exosome miR-215-5p to Prevent Necroptosis of Neuronal Cells and a Model for Early Diagnosis of Alzheimer’s Disease

**DOI:** 10.3389/fnagi.2022.860364

**Published:** 2022-05-09

**Authors:** Yisheng Chen, Yaying Sun, Zhiwen Luo, Jinrong Lin, Beijie Qi, Xueran Kang, Chenting Ying, Chenyang Guo, Mengxuan Yao, Xiangjun Chen, Yi Wang, Qian Wang, Jiwu Chen, Shiyi Chen

**Affiliations:** ^1^Department of Sports Medicine, Huashan Hospital, Fudan University, Shanghai, China; ^2^Shanghai Jiao Tong University School of Medicine, Shanghai Jiao Tong University, Shanghai, China; ^3^Department of Orthopaedics, The Second Affiliated Hospital, Zhejiang University School of Medicine, Hangzhou, China; ^4^Department of Orthopedics, Shanghai General Hospital, Shanghai Jiao Tong University School of Medicine, Shanghai Jiao Tong University, Shanghai, China; ^5^Department of Orthopaedic Surgery, The Third Hospital of Hebei Medical University, Shijiazhuang, China; ^6^Key Laboratory of Biomechanics of Hebei Province, Orthopaedic Research Institution of Hebei Province, Shijiazhuang, China; ^7^Huashan Hospital, Fudan University, Shanghai, China; ^8^Department of Central Laboratory, The Affiliated Taian City Central Hospital of Qingdao University, Tai’an, China

**Keywords:** Alzheimer’s disease, exercise, necroptosis, exosomes, miR-215-5p, neural network prediction model

## Abstract

Exercise is crucial for preventing Alzheimer’s disease (AD), although the exact underlying mechanism remains unclear. The construction of an accurate AD risk prediction model is beneficial as it can provide a theoretical basis for preventive exercise prescription. In recent years, necroptosis has been confirmed as an important manifestation of AD, and exercise is known to inhibit necroptosis of neuronal cells. In this study, we extracted 67 necroptosis-related genes and 32 necroptosis-related lncRNAs and screened for key predictive AD risk genes through a random forest analysis. Based on the neural network Prediction model, we constructed a new logistic regression-based AD risk prediction model in order to provide a visual basis for the formulation of exercise prescription. The prediction model had an area under the curve (AUC) value of 0.979, indicative of strong predictive power and a robust clinical application prospect. In the exercise group, the expression of exosomal miR-215-5p was found to be upregulated; miR-215-5p could potentially inhibit the expressions of *IDH1*, *BCL2L11*, and *SIRT1*. The single-cell SCENIC assay was used to identify key transcriptional regulators in skeletal muscle. Among them, *CEBPB* and *GATA6* were identified as putative transcriptional regulators of miR-215. After “skeletal muscle removal of load,” the expressions of *CEBPB* and *GATA6* increased substantially, which in turn led to the elevation of miR-215 expression, thereby suggesting a putative mechanism for negative feedback regulation of exosomal homeostasis.

## Introduction

Alzheimer’s disease (AD), a global health concern, is a neurodegenerative disease whose pathology is thought to be characterized by neurofibrillary tangles due to extracellular β-amyloid deposition and tau hyperphosphorylation (p-tau). At present, as the pathogenesis of AD remains unclear, no effective treatment is available (Lei et al., 2021). Recent studies suggest that physical activity is an important preventative method for AD and can greatly improve the quality of life of the patient (Dimities et al., 2021). Exercise prescription has now been included in the agenda (Luan et al., 2019). However, further research is needed to determine and identify patients at risk for AD who may require early exercise prescription. The specific mechanisms underlying exercise-related prevention of AD are complex and remain largely unclear (De la Rosa et al., 2020). Therefore, the construction of an accurate AD risk prediction model may serve as the basis for early implementation of the exercise prescription therapy (Li et al., 2016).

In recent years, necroptosis has been identified as an important player in neurodegenerative diseases, including AD (Caccamo et al., 2017; Yuan et al., 2019). Necroptosis, also called programmed necrosis, is a regulated mode of necrotic cell death mediated by RIP1 and RIP3 kinases. In 2021, Fu et al. (2021) reported that exercise could help prevent necroptosis of cardiomyocytes. Accumulating evidence indicates that exercise can prevent necroptosis through various pathways (Zhang et al., 2022). However, to the best of our knowledge, at present, the number of mechanistic studies on exercise-based prevention of necroptosis in AD is scarce. The study of necroptosis-related expression epistasis may help in constructing a new exercise prescription-sensitive AD risk prediction model.

In what ways might exercise prevent necroptosis in AD patients? Recent findings suggest that exosomes are mediators of systemic adaptation to endurance exercise, that is, they are important channels through which exercise may regulate other tissues (Safdar and Tarnopolsky, 2018). Exosomes can cross the blood–brain barrier and influence the development and progression of AD (Jiang et al., 2019; Soares Martins et al., 2021). Exercise has the potential to prevent the onset of neurodegeneration by modulating changes in exosome levels in the plasma (Fuller et al., 2020). We hypothesized that miRNAs carried by the exercise-regulated exosomes may affect AD development by inhibiting the necroptosis-related pathways, while lncRNAs exert a competitive effect by repressing these miRNAs (Tay et al., 2014). Recent studies show that lncRNAs play an important role in the process of necroptosis (Su et al., 2016; Jiang et al., 2021). Therefore, evaluating necroptosis-associated lncRNAs is necessary. Analysis of single-cell sequencing data yields transcriptional regulatory relationships in the organization of the locomotor system at single-cell resolution, thereby providing a plausible explanation for the differential expression of miRNAs in blood as a result of the exercise (Holland et al., 2020; Chen Y. et al., 2021; Wen-Jin et al., 2021). Therefore, analysis of scRNA transcriptional features of skeletal muscles is beneficial to understand the potential mechanisms and regulatory networks of miRNA upregulation in exosomes after exercise. This study aimed to construct a predictive model for the risk of AD associated with necroptosis in an effect to provide a theoretical basis for early administration of exercise prescription. We also examined the potential mechanisms underlying exosomal miRNA expression and regulation after exercise; these findings are expected to provide a potential biological basis for preventative exercise prescription for pre-AD patients.

## Materials and Methods

### Data Acquisition and Variance Analysis

A total of five datasets were downloaded from the GEO database^1^, namely, two AD datasets (GSE33000 and GSE44770), a dataset consisting of altered microRNA expressions in circulating blood after exercise (GSE144627), a skeletal muscle load-related dataset (GSE155933), and a single-cell transcriptome dataset of the skeletal muscle (GSE138826) (Supplementary Table 3). The GSE33000 dataset is based on the GPL4372 platform. This was examined on the Rosetta/Merck Human 44k 1.1 microarray. In this study, 467 human brain tissues were selected; among them, 310 were specimens from AD patients and 157 were specimens from non-demented controls. All brain tissue samples were obtained from the Harvard Brain Tissue Resource Center (HBTRC) (Narayanan et al., 2014). The GSE44770 dataset is also based on the GPL4372 platform. This was examined on the Rosetta/Merck Human 44k 1.1 microarray. A total of 230 human brain tissues were included; among them, 129 specimens were obtained from AD patients and 101 specimens were obtained from non-demented controls (Zhang et al., 2013). The GSE144627 dataset is based on the GPL15520 platform. The miRNA samples of circulating exosomes from 10 older adults (five endurance trainers and five sedentary individuals) were analyzed using the Illumina MiSeq (Homo sapiens) system (Nair et al., 2020). The GSE155933 dataset is based on the GPL24047 platform. This was assayed using the Affymetrix Human Transcriptome Array 2.0. A total of 230 skeletal muscle samples were included and the dataset comprised information on muscle conditions before and after muscle resistance training/unloading (Timmons et al., 2019). The GSE138826 dataset, based on the GPL24247 platform, was screened using Illumina NovaSeq 6000. It is a single-cell dataset from the mouse tibialis anterior muscle tissue (Oprescu et al., 2020). Differential expression analysis was performed using the limma package in R software; the fold change (FC) and false discovery rate (FDR) of all differential genes were noted, and a *p*-value < 0.05 was the criterion for statistical significance (Costa-Silva et al., 2017).

### Correlation Analysis for Genes and Long Non-coding RNA Associated With Alzheimer’s Disease and Necroptosis

The necroptosis gene set, M24779.gmt, consists of eight necroptosis genes, all of which were downloaded from the Gene Set Enrichment Analysis (GSEA)^2^ database. Finally, 67 necroptosis-related genes were included in the study (Zhao et al., 2021). After Pearson correlation analysis for all lncRNAs and the 67 necroptosis-related genes in the gene expression matrix, a total of 32 lncRNAs were identified and defined as significant necroptosis-related lncRNAs in AD (Pearson correlation coefficients > 0.4 and *p* < 0.001).

### Random Forest Analysis and Neural Network Model

To ensure the reproducibility of the results, we set the seed at “123456.” The random forest analysis was performed using the “randomForest” package in the R software. The parameters were set using the default function with 500 “trees.” Since the error of the random forest model was minimized when 324 trees were used, accordingly, the importance of each gene was defined. The neural network model was constructed using the “neuralnet” and “NeuralNetTools” packages, with the seed set to “12345678” and the parameter to “hidden = 5.” The GSE33000 dataset was used as the training group, and the GSE44770 dataset was used as the test group. The “pROC” package was used to plot the receiver operating characteristic (ROC) curve for the model.

### Intersection Analysis and the Construction of the miRNA–mRNA Regulatory Network

The visualization function in R software was used to draw the Venn diagram. Prediction of mRNA targets for corresponding miRNAs was based on the database results. miRNA/mRNA interactions were identified using the miRWalk database^3^; subsequently, the miRNA–mRNA regulatory networks were constructed.

### SCENIC Data Analysis and Regulatory Gene Network Based on the scRNA-Seq Analysis

The scRNA-seq analysis was performed using the “Seurat” package. Briefly, the cell clustering and clustering for the GSE138826 dataset followed the methods described previously by Oprescu et al. (2020). SCENIC supports the analysis of positive transcriptional regulation, which implies that it can screen transcription factors that regulate miRNAs during positive transcription. An improved version of the SCENIC method was used to screen the key transcription factors in skeletal muscles, as described previously (Suo et al., 2018; Chen Y. et al., 2021; Lin et al., 2021a,b). Moreover, to quantify the cell-type specificity of a regulon, we adapted an entropy-based strategy that was previously used for the analysis of gene expression data (Cabili et al., 2011).

### Immune Infiltration and Immune Checkpoint Analysis

As an extension of the GSEA, a single sample gene set enrichment analysis (ssGSEA) allows for defining an enrichment score corresponding to the absolute enrichment of a gene set for each sample within a given dataset. This was published in 2009 (Barbie et al., 2009). The “GSVA” and “GSEABase” packages in the R software were used for immuno-infiltration analysis; the “limma” package was used for differential analysis, and the results of the final analyses were visualized using the “ggpubr” package. Immune checkpoint-related genes were extracted to screen the differentially expressed immune checkpoint genes in AD.

### Gene Set Enrichment Analysis

The R (version 3.6.3) software was used for GSEA and visualization, as described in previous studies (Wang et al., 2020). The reference gene collection “c2.cp.v7.2.symbols.gmt” and the species *Homo sapiens* were chosen for this study, and the analysis was carried out according to the clusterProfiler package’s instructions. Pathways meeting a FDR < 0.25 and p.adjust < 0.05 were considered significantly enriched.

### Protein–Protein Interaction Network and Drug Sensitivity Analysis

The STRING database^4^ was used to calculate the level of interaction likelihood and construct the network of interactions between known and predicted proteins. The more complex was the protein-protein relationship network encoded by a gene, the more was its importance for the gene function. The protein–protein interaction (PPI) network encoded by DEGs was constructed using STRING, and the key genes were screened. Drug sensitivity analysis was based on the results of the “pRRophetic” package; the “limma” package was used for variance analysis. The “pRRophetic” package uses the expression matrix and drug handling information from the Cancer Genome Project (CGP), a database comprising 138 anticancer drugs tested against 727 cell lines. The “cgp2016” dataset was used for the bulk prediction of drug data.

### Statistical Methods and Neural Network-Based Clinical Prediction Model

Based on the neural network prediction model, we constructed a new logistic regression-based AD risk prediction model in order to provide a visual basis for the formulation of exercise prescription. Factors with *p*-values < 0.05 were further screened and used to construct a column line graph prediction model, as described previously (Chen et al., 2020; Ying et al., 2021; Zhou et al., 2021). The GSE44770 dataset was used as an external dataset to validate the findings. These data were used for principal component analysis (PCA) to assess their ability to discriminate between different outcomes as our previous research (Ying et al., 2021). Calibration curves were plotted to evaluate the accuracy of the nomogram. Moreover, the C-index and AUC values were also calculated for further verification of the prediction accuracy. Besides, accuracy, *F*-value, precision, and recall of each dataset and proposed nomogram were also described as our previous research (Wang et al., 2020). Finally, decision curves were used to evaluate the clinical applicability of the nomogram (Kang et al., 2020).

## Results

### Screening Necroptosis-Associated Messenger RNA and Long Non-coding RNA in Brain Tissue From Alzheimer’s Disease Patients

**FIGURE 1:**
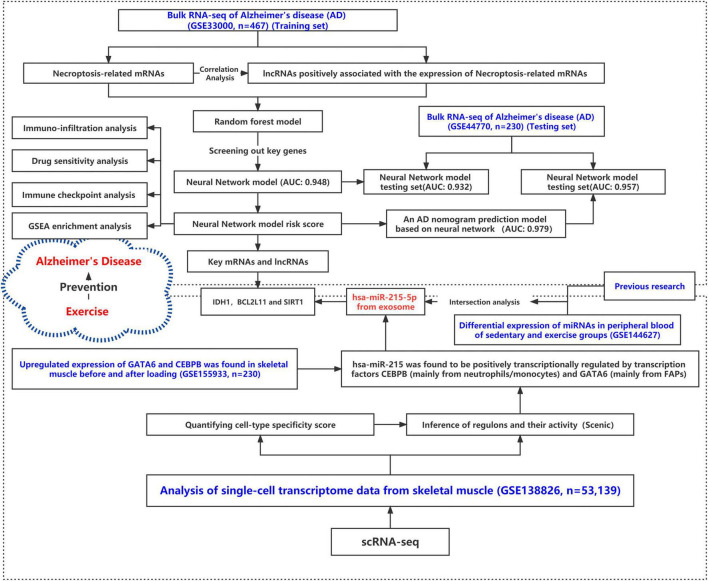
Research roadmap for this study.

**FIGURE 2:**
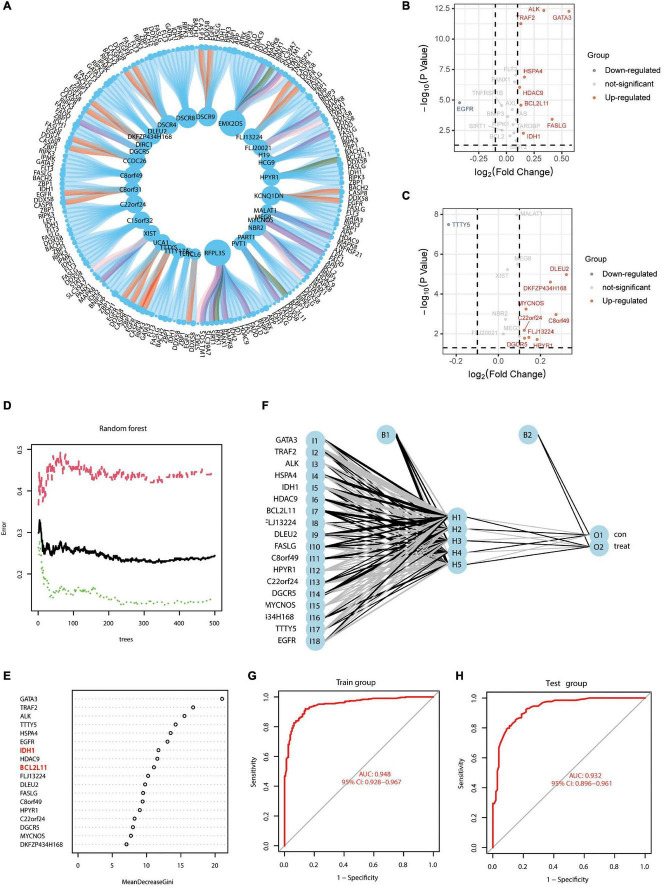
Screening of necroptosis-associated mRNAs and lncRNAs in AD brain tissue to construct AD risk score scores. **(A)** Circle diagram of necroptosis-related lncRNAs–mRNAs correlation analysis; lncRNAs are in the middle of the circle, and mRNAs are in the outer periphery of the circle. Linkage of peripheral circle origin to inner circle origin indicates a significant correlation. Red connecting lines indicate that these mRNAs are highly expressed in AD, and purple indicates that these mRNAs are lowly expressed in AD. **(B)** Volcano map of necroptosis-related mRNAs differentially expressed in AD. **(C)** Volcano map of necroptosis-related lncRNAs differentially expressed in AD. **(D)** Prediction error diagram of random forest model; green line indicates training group error, red line indicates test group error and black line indicates overall group error. **(E)** Importance analysis of genes in predicting AD in random forest, filtering all genes with scores greater than 2 according to the model with the lowest error point in panel **(D)**. **(F)** Schematic diagram of a neural network model for AD diagnosis prediction based on gene expression features. **(G,H)** ROC curves of the AD neural network prediction model for the training group (AUC = 0.948; 95% CI: 0.928–0.967) and the test group (AUC = 0.932; 95% CI: 0.896–0.961).

The research flow schema of this study is shown in Figure 1. Sixty-seven necroptosis-related genes were initially included (Zhao et al., 2021). After Pearson correlation analysis for all lncRNAs in the GSE33000 gene expression matrix and 67 necroptosis-related genes, a total of 32 lncRNAs were identified for subsequent analysis. These lncRNAs were considered as significant necroptosis-associated lncRNAs (Pearson correlation coefficients > 0.4 and *p* < 0.001) in AD. The PPI network for these lncRNAs is positively associated with mRNAs as shown in Supplementary Figure 1. Our main objective was to screen lncRNAs that may regulate exosomal microRNAs. MicroRNAs negatively regulate mRNAs. Therefore, based on the ceRNA theory, we only screened the lncRNAs–mRNA pairs, wherein the lncRNAs were positively associated with mRNAs. Figure 2A presents a circle diagram of necroptosis-related lncRNAs–mRNAs relationship pairs. The lncRNAs are in the middle of the circle; mRNAs are in the outer periphery of the circle, and the linkage of the outer circle origin with the inner circle origin indicates a significant correlation. To determine whether these lncRNAs–mRNAs relationship pairs are differentially expressed in AD patients and normal patients, we performed the difference analysis. Red connecting lines indicate that these mRNAs are highly expressed in AD, and purple indicates that these mRNAs are lowly expressed in AD. Volcano maps were used to visualize the significantly different lncRNAs and mRNAs. Since data on microRNAs in the GSE33000 dataset are lacking, the dataset of microRNAs is not shown in the volcano plot. The volcano plots for the differentially expressed genes between AD and normal brain tissues are shown in Figures 2B,C. *GATA3*, *ALK*, *TRAF2*, *HSPA4*, *HDAC9*, *BCL2L11*, *FASLG*, and *IDH1* are significantly overexpressed in AD patients. Also, the EGFR expression is downregulated in AD patients. *DLEU2*, *DKFZP434H168*, *MYCNOS*, *C22or24*, *C8orf49*, *FLJ13224*, *DGOR5*, and *HPYR1* are lncRNAs that are also overexpressed in AD.

### Neural Network Model Based on Random Forest Analysis to Evaluate the Alzheimer’s Disease Risk Score

Genes showing significant differences were used for analysis using the random forest model. In Figure 2D, the green line indicates the training group error; the red line indicates the test group error, and the black line indicates the overall group error. We found that the random forest model exhibited the lowest error at 324 “trees,” and all genes with scores less than 2 were excluded (Figure 2E). Due to the large error, it was necessary to further optimize the random forest model. The ROC curves for the AD neural network prediction model (Figure 2F) are shown in Figures 2G,H; the AUC of the training group was 0.948 (95% CI: 0.928–0.967) and that of the test group was 0.932 (95% CI: 0.896–0.961). This result suggested that mRNAs and lncRNAs based on necroptosis-related genes show good predictive ability.

### Alzheimer’s Disease Risk Assessment Model Based on the Neural Network

**FIGURE 3:**
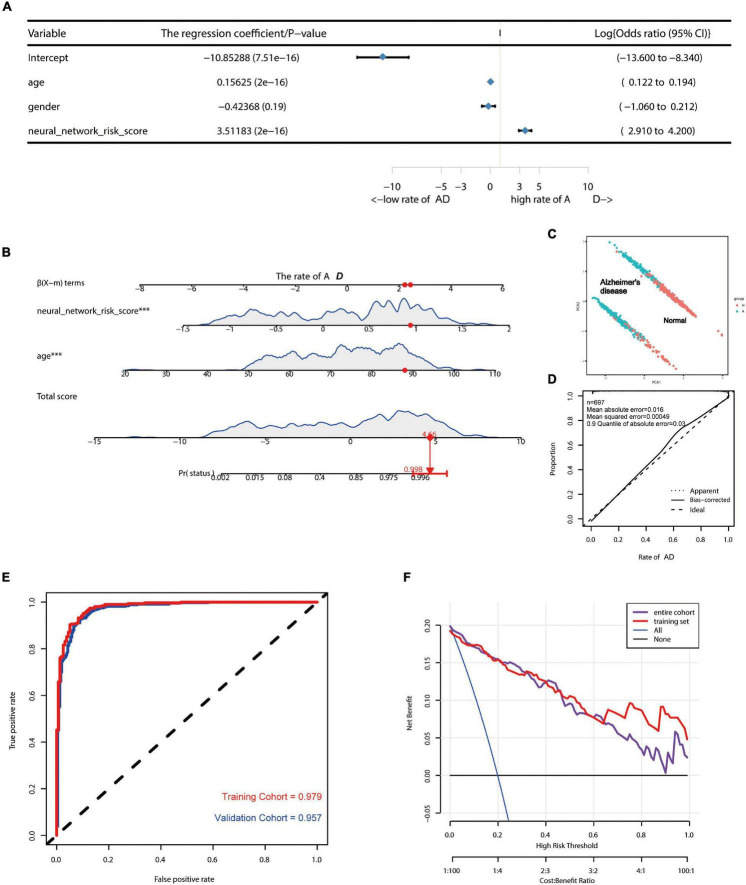
Construction of AD risk nomogram model based on neural network model. **(A)** A forest plot for multifactor logistical analysis based on age, gender, and neural network risk score. **(B)** A nomogram prediction model was constructed using neural network risk score and age. **(C)** PCA suggests that the model is better able to distinguish AD from normal patients. **(D)** The calibration curve of this nomogram prediction model after including a sample of 697 cases. **(E)** ROC curves of the AD risk nomogram prediction model constructed based on the neural network model for the training group (AUC = 0.979) and the test group (AUC = 0.957). **(F)** DCA curves of this nomogram prediction model.

Since this neural network model did not include basic clinical features such as age and gender, we constructed a forest plot using the age, gender, and neural network risk score (Figure 3A). A forest plot for the multifactor logistical analysis based on age, gender, and neural network risk scores is shown in Figure 3A. The *p*-values for multifactor regression analysis of age and neural network risk score were significant and were subsequently used to construct the nomogram prediction model (Figure 3B). A PCA suggested that the model could better distinguish AD from normal patients (Figure 3C). The calibration curve was plotted using data from the entire group (Figure 3D). The mean absolute error of this calibration curve was 0.016, while the mean squared error was 0.0049 (Figure 3D). To determine the prediction accuracy of this neural network model, we calculated the AUC for the ROC curve (Figure 3E). The AUC value of the training group was 0.979, while that of the test group was 0.957. In addition, the C-index was calculated and reached 0.979 [95% credibility interval (95% CI; 0.959–0.983)] in the training set and 0.957 (95% CI; 0.968–0.990) in the validation set (Table 1). Besides, we also described the accuracy, *F*-value, precision, and recall of each dataset and proposed nomogram in Table 2. This indicated that the prediction model was capable of accurately representing the transcriptomic aspects of the brains of AD patients and had high predictive potential. The DCA curve for this nomogram prediction model is shown in Figure 3F, which suggests that the model has a very high predictive power.

**TABLE 1:** C-index of the prediction model.

Dataset group	C-index of the prediction model	
	C-index	The C-index (95% CI)
Training set	0.979	0.959–0.983
Validation set	0.957	0.968–0.990
Entire cohort	0.97	0.930–0.984

**TABLE 2:** Accuracy, *F*-value, precision, and recall of each dataset.

	Dataset group		
	Entire cohort	Training set	Validation set
Accuracy	0.9283	0.9358	0.9087
*F*-value (α = 1)	0.9433	0.9518	0.9195
Precision	0.9391	0.9487	0.9091
Recall	0.9476	0.9548	0.9302

### Correlation Analysis for Alzheimer’s Disease Risk Assessment Models With Local Immune Microenvironment and Drug Sensitivity

**FIGURE 4:**
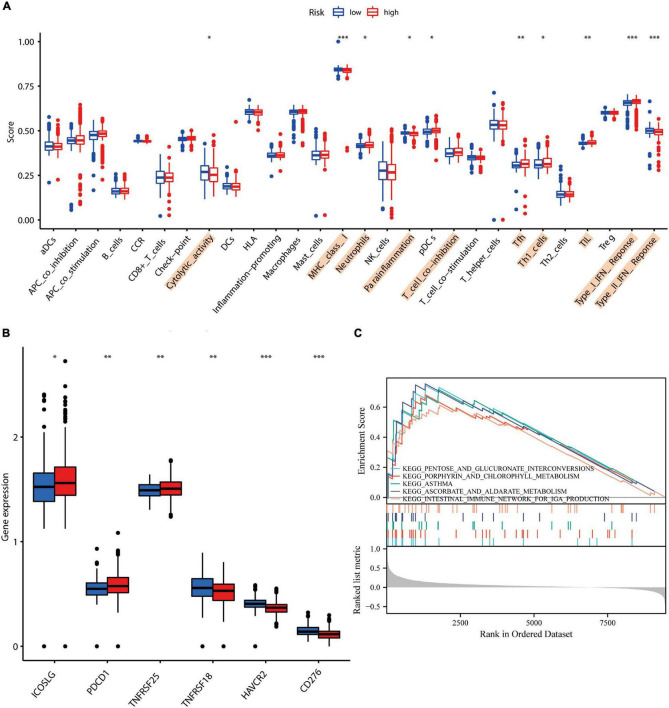
Correlation study of AD risk score scores with local immune characteristics. **(A)** Immune cell infiltration analysis based on ssGSEA. **(B)** Correlation of high and low AD risk scores with immune checkpoints. **(C)** GSEA enrichment analysis based on AD scores. * represents *p*-value < 0.05, ^**^ represents *p*-value < 0.01, and ^***^ represents *p*-value < 0.001.

Previous studies show that microenvironmental alterations characterized by an imbalance in the immune microenvironment of the brain induced upon damage to the blood–brain barrier are closely related to the AD pathogenesis (Lu et al., 2021). In our investigation, using ssGSEA, we determined the degree of immune cell infiltration. Cytolytic activity, MHC class I, neutrophils, para-inflammation, T cell co-inhibition, Tfh, TIL, Type I, and Type II IFN responses were all found to be significantly associated with the AD risk assessment model (Figure 4A). ICOSLG, PDCD1, and TNFRSF25 were found to be overexpressed in patients in the high-risk group. In the high-risk group, the expressions of TNFRSF18, HAVCR2, and CD276, were found to be downregulated (Figure 4B). Findings from GSEA revealed a substantial association between the gut immune network for IgA production and the AD risk score (Figure 4C). The aforementioned results indicated that this model of AD risk assessment was intimately connected to the local immunological microenvironment. Imbalances in the circulatory system and intracerebral immune microenvironment were found to be strongly related to neuronal necroptosis. Additionally, Supplementary Figure 2 illustrates the differential susceptibility of neuronal cells to medicines in individuals at high and low risk of developing AD. Thus, those medicines may contribute to AD by impairing neuronal necroptosis.

### Upregulation of Exosomal hsa-miR-215 Expression in Circulating Blood After Exercise May Contribute to the Inhibition of Alzheimer’s Disease Necroptosis-Related Genes

**FIGURE 5:**
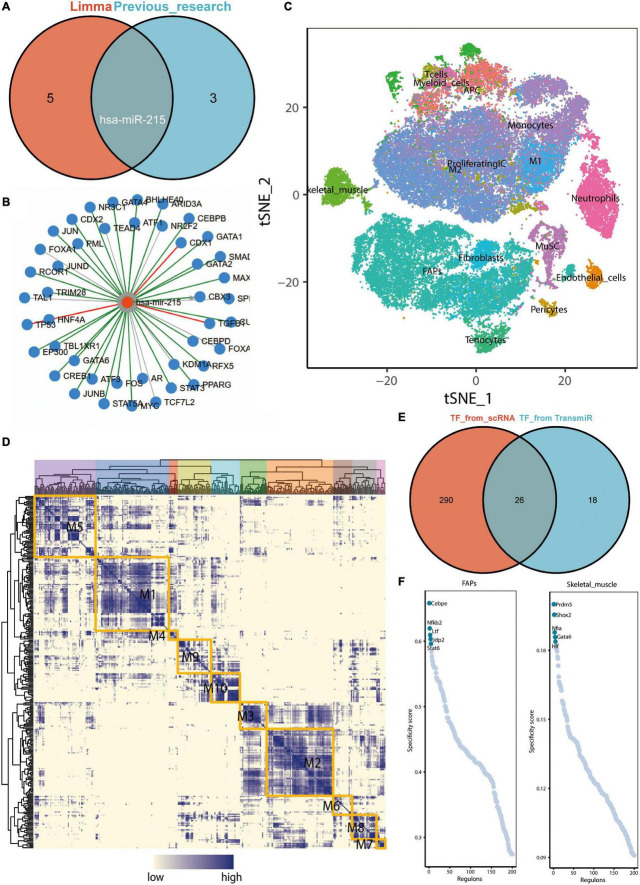
Upregulation of exosome hsa-miR-215 expression in circulating blood after exercise and analysis of key transcription factors in skeletal muscle. **(A)** Venn diagram demonstrating that miR-215 is the microRNA found to be co-differentially expressed in previous studies and in the GSE144627 dataset. The “limma” here refers to the difference analysis for the GSE144627 dataset. **(B)** Prediction of upstream transcription factors that may regulate hsa-miR-215 transcription based on TransmiR v2.0. **(C)** tSNE distribution map of GSE138826 single cell dataset. **(D)** Regulatory modules identified based on the connection specificity index (CSl) matrix. **(E)** Venn diagram showing key transcription factors from skeletal muscle single cells (*n* = 316) with possible common transcription factors predicted to regulate miR-215 based on the TransmiR database (*n* = 44). **(F)** Ranking of regulators of FAPs and skeletal muscle based on regulatory specificity score (RSS).

Previous studies suggest that regular exercise leads to the upregulation of three microRNAs, namely, miR-486-5p, miR-215-5p, and miR-941, in the peripheral blood, while that of the exosomal miR-151b is downregulated (Nair et al., 2020). We performed a differential analysis using the GSE144627 dataset and found that miR-1306-3p, miR-215-5p, miR-432-5p, miR-129-5p, miR-370-3p, and miR-197-3p were all upregulated in the exercise group (Supplementary Table 1). The logFC values for miR-215-5p and miR-197-3p expressions were greater than 2. The Venn diagram demonstrated that the microRNA, miR-215-5p, was co-significantly differentially expressed in both previous studies and the GSE144627 dataset (Figure 5A). The miRWalk database was used to construct a miRNA–mRNA regulatory network (Supplementary Table 2). We reasonably hypothesized that the upregulation of exosomal hsa-miR-215 expression in circulating blood after exercise may contribute to the inhibition of AD necroptosis-related genes (e.g., *IDH1*, *SIRT1*, and *BCL2L11*). Among them, *SIRT1* and *BCL2L11* have been previously validated as the target genes of miR-215-5p.

### CEBPE and *GATA6* Were Defined as Potential Transcriptional Regulators Leading to the Upregulation of hsa-miR-215 Expression

Based on the TransmiR v2.0 database, a total of 44 TFs, the possible upstream transcriptional regulators regulating hsa-miR-215 transcription, were predicted (Figure 5B). Cell clustering in the GSE138826 dataset was performed based on the findings of Oprescu et al. (2020; Figure 5C). Subsequently, the connection specificity index (CSl) matrix was constructed using the SCENIC method, and a total of ten modules were obtained (Figure 5D). In these modules, 316 key TFs were included. The Venn diagram shows the intersection of key TFs from skeletal muscle scRNA-seq data with those predicted to potentially regulate miR-215 using the TransmiR database (Figure 5E). The regulator rankings for FAPs and skeletal muscle based on the Regulon specificity score (RSS) are shown in Figure 5F. Thus, Cebpe and GATA6 were identified as key TF in FAPs and skeletal muscle cells, respectively. Since Cebpe and GATA6 could potentially regulate miR-215, we speculated that the upregulation of Cebpe and GATA6 expressions in skeletal muscles could elevate miR-215 levels in exosomes.

### Upregulation of *CEBPE* and *GATA6* Expression After Unloading Muscle Load May Help Prevent the Excessive Downregulation of miR-215

**FIGURE 6:**
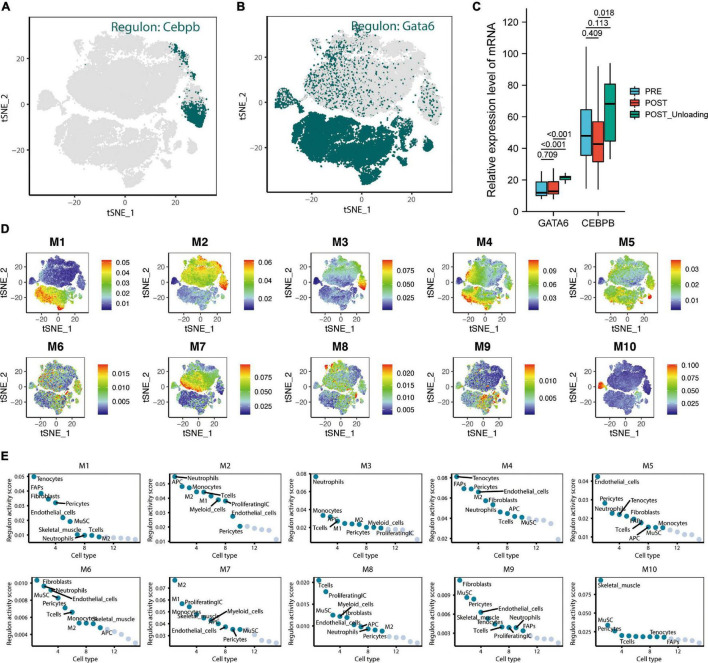
CEBPE and *GATA6* were defined as potential transcriptional regulators leading to the upregulation of hsa-miR-215 expression and analysis of skeletal muscle single-cell transcriptional regulatory module. **(A)** tSNE plot demonstrating the expression characteristics of regulon *CEBPB* in single cells. **(B)** The tSNE plot demonstrates the expression characteristics of regulon *GATA6* in single cells. **(C)** Expression characteristics of *GATA6* and *CEBPB* in the pre-loading (PRE), post-loading (POST), and de-loading groups (POST Unloading) in the GSE155933 dataset, with *p*-values < 0.05 defined as significant differences. **(D)** The average expression characteristics of genes in each module are shown on the tSNE plot. **(E)** Plot of ranking distribution of individual cells in each module according to the regulon activity score.

**FIGURE 7:**
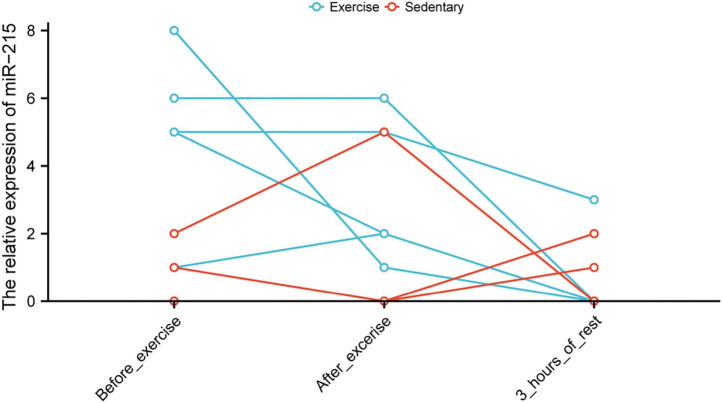
Altered levels of miR-215 in circulating blood exosomes before and after exercise.

The tSNE plot was used to demonstrate the expression characteristics of the regulon, *CEBPB*, and *GATA6* from the scRNA-seq data (Figures 6A,B). *GATA6* and *CEBPB* were found to be significantly upregulated in the de-load group (Figure 6C). The tSNE plot shows the average expression characteristics of each gene module (Figure 6D). The distribution of the rankings of each cell type in each module according to the regulon activity score is also shown (Figure 6E). This suggested that *CEBPB* may exert an important transcriptional regulatory effect in the M2 and M3 cell clusters, while *GATA6* may play a critical transcriptional regulatory role in the M4 and M5 cell clusters. *CEBPB* and *GATA6* were found to be significantly upregulated after muscle exercise and de-loading. In a previous study, we found that regular exercise resulted in a significant increase in baseline expression of exosomal microRNA-215. However, acute exercise did not elicit the same effect on exosomal miRNA-215 expression (Figure 7). These suggest that the upregulation of miR-215 expression is not transient, rather it is the result of longterm exercise treatment. Although further validation is needed, we hypothesize that the transcriptional activity of CEBPB and GATA6 in upregulating miR-215 may be a potential negative regulatory feedback mechanism in exosome homeostasis after skeletal muscle unloading.

## Discussion

Exercise promotes longevity and fitness. It is also used therapeutically to prevent and treat several physical disorders, including cardiovascular diseases and diabetes. Although exercise offers numerous benefits for mood, learning ability, spatial and verbal memory, and cognition, the molecular mechanisms underlying these benefits remain unclear. Exercise helps in the prevention and treatment of disorders associated with age-related cognitive loss, such as AD and dementia (Lautenschlager et al., 2019).

Based on necroptosis-related genes, we constructed an AD risk prediction model with high predictive accuracy, excellent predictive power, and good prospects for clinical application. In exosomes, miR-215-5p could potentially target some genes involved in necroptosis (e.g., *IDH1*, *BCL2L11*, and *SIRT1*). Additionally, the expressions of *CEBPB* and *GATA6* were highly upregulated following exercise and load decrease. We postulated that the elevation in miR-215 expression through the overexpression of *CEBPB* and *GATA6* may serve as a negative feedback loop for the regulation of exosomal homeostasis following a decrease in the skeletal muscle load.

First, we screened the AD brain tissue data for necroptosis-associated mRNAs and lncRNAs. This is the first study to test a neural network model for predicting AD risk using necroptosis-associated miRNAs and lncRNAs. This prediction model exhibited greater predictive power than some previously described models. For instance, the prediction model of Chen W. et al. (2021) has an AUC of 0.872, while that proposed by Wang et al. (2021) has an AUC of 0.822. The C-index of the AD prediction model combining radiomic-clinical-laboratory data in the study by Tang et al. (2021) was also lower than that predicted using the model constructed in this study (0.979; 95% CI, 0.959–0.983); the maximum was at 0.950 (95% CI, 0.929–0.971). This suggested that the clinical features of AD patients could be well predicted based on necroptosis-related genes.

*DLEU2, DKFZP434H168, MYCNOS, C22or24, C8orf49, FLJ13224*, and *DGOR5* are also overexpressed in AD. They have all been incorporated into neural network models as risk predictors of AD. To the best of our knowledge, this study is the first to reveal a potential relationship between these lncRNAs and AD. Interestingly, in our previous study, lncRNA DLEU2 acts as a miR-181a sponge to regulate SEPP1 and inhibit skeletal muscle differentiation and regeneration (Wang et al., 2020). Combining the results of the previous study and this study, we suggested that DLEU2 is a potential common risk factor for the development of sarcopenia and Alzheimer’s disease. Therefore, DLEU2 was proposed as a risk marker for sarcopenia and Alzheimer’s disease in this study.

In addition, the AD risk assessment model was closely associated with the local immune microenvironment and the pathways in this study, consistent with previous findings. Cytolytic activity-related proteins are associated with neurodegenerative diseases (Tampio et al., 2020). Higher neutrophil counts are associated with AD (Ramos-Cejudo et al., 2021). The IFN pathway is significantly upregulated in AD and correlates significantly with disease severity and complement activation pathway (Roy et al., 2020). Immune checkpoints are also associated with the development of AD. In this study, the immune checkpoints, such as ICOSLG, PDCD1, TNFRSF25, TNFRSF18, HAVCR2, and CD276, were found to be significantly associated with AD risk scores (Baruch et al., 2016). Moreover, GSEA showed that immune-related pathways were closely associated with the AD risk assessment model. Therefore, the regulation of the immune system on exercising may be an important mechanism underlying its preventative effects against the development of AD (Madore et al., 2020).

Overall, changes in the expression of miRNAs enriched in muscle exosomes after exercise are consistent with changes in expression produced due to long-term regular muscle exercise (Nair et al., 2020). Exosomal miR-215-5p was upregulated in the exercise group, consistent with the results of a previous study (Nair et al., 2020). However, acute exercise was not found to have a significant effect on the expression of exosomal microRNA-215-5p. In this research, the miRWalk database was used to construct a miRNA–mRNA regulatory network. Based on the regulation principle of ceRNA, the upregulation of exosomal hsa-miR-215 expression in circulating blood after exercise was found to potentially contribute to the inhibition of AD necroptosis-related genes such as *IDH1*, *SIRT1*, and *BCL2L11*. According to miRWalk database, *SIRT1* and *BCL2L11* have been previously validated as the target genes of miR-215-5p. Therefore, miR-215-5p may target and bind *IDH1*, *BCL2L11*, and *SIRT1* mRNAs, thereby inhibiting their protein synthesis. *IDH1, BCL2L11*, and *SIRT1* have been revealed to be necroptosis-related genes in previous studies, and they play important functions in the development of necroptosis (Zhao et al., 2021). For example, IDH1/2-driven tumorigenesis was found to be associated with necroptosis (Schmauss et al., 2017). Targeted inhibitors of SIRT1 can downregulate necroptosis pathway activity by inhibiting the function of SIRT1 (Nikseresht et al., 2019). In skeletal muscle, the myoprotective effects of unacylated ghrelin on pressure-induced tissue injury were associated with SIRT1 and necroptosis signaling (Ugwu et al., 2017). Furthermore, BCL2L11 was also found to play an important function in the necroptosis pathway (Bohgaki et al., 2011; Cabon et al., 2012; Locatelli et al., 2014). However, to the best of our knowledge, the effect of exercise on *IDH1* and *BCL2L11* expression has not been studied in detail. In AD patients, high *SIRT1* expression has been suggested to exert neuroprotective effects for preventing the occurrence of neuronal apoptosis (Gomes et al., 2018). Exercise can upregulate the AMPK-SIRT1-TFEB signaling pathway, thereby activating the lysosomal functions in the brain (Huang et al., 2019). Immune checkpoints are associated with the development of AD. In this study, the immune checkpoints, such as ICOSLG, PDCD1, TNFRSF25, TNFRSF18, HAVCR2, and CD276, were found to be significantly associated with AD risk scores. The result from the miRWalk database analysis suggested that miR-215-5p could target and inhibit *BCL2L11* expression to a certain extent. Downregulation of *BCL2L11* expression can prevent the development of AD (Shi et al., 2016; Li et al., 2020). Therefore, “miR-215-5p targeting *BCL2L11*” may be an important potential mechanism underlying the prevention of AD occurrence.

The levels of expression of *CEBPB* and *GATA6* were considerably elevated following muscle load decrease. To date, no study reports the association between exercise and *GATA6* expression. *CEBPB* is implicated in myocardial protection, and its deficiency results in cardiomyocyte hypertrophy and proliferation (Boström et al., 2010). In addition, endurance exercise training can affect the expression of *CEBPB* (Walton et al., 2019). Interestingly, *CEBPB* expression in blood is consistent with the CEBPB-dependent muscle repair process, suggesting that *CEBPB* may be a potential marker for muscle repair (Blackwell et al., 2015). The upregulation of miR-215 expression brought about by the elevated expressions of *CEBPB* and *GATA6* after muscle loss of load may be the potential mechanism underlying the negative feedback regulation of exosomal homeostasis after skeletal muscle loss of load. However, the mechanisms involved need to be further investigated.

This study elucidated a possible mechanism by which exercise protects against the development of AD by modifying the aspects of circulating blood exosomes. miR-215-5p in exosomes may exert protective effects against the development of AD by targeting *BCL2L11*. In this study, a training group of more than 400 cases and a test group of more than 200 cases were obtained using two different datasets, thus, potentially making the results of this study highly reliable. The high AUC may be attributed to the role of necroptosis in the development of AD. In addition, the construction of the logistic prediction model based on the neural network model may have helped in improving the prediction accuracy of this study. Although the sample size used for this investigation was sufficient, the findings must be validated in clinical trials. Additionally, the contradictory outcomes of the data analysis, while interpretable, require further verification using clinical data. We proposed some necrotizing apoptosis-related biomarkers associated with AD. However, we did not analyse the association of these markers with exercise due to objective constraints. In the future, single-cell transcriptome sequencing data before and after exercise should also be made available. Although the predictive effect of the model was high, the prediction results still seemed to be somewhat biased from the PCA. This study was focused on constructing a prediction model based on necroptosis-related characteristics. However, the occurrence of AD is influenced by several other factors, which may affect the prediction results to some extent. Furthermore, the prediction model in this study is more similar to a diagnostic model. The progressive nature of the disease leads to a progressive change in the disease marker as well. This feature permits the potential diagnostic use of the markers in the discriminant model as tools for staging or evaluating prognosis. This means that the higher the risk score, the higher the risk for future diseases. Therefore, it follows that patients with higher risk scores are more likely to benefit from exercise prescription.

Based on the neural network prediction model, we constructed a new logistic regression-based AD risk prediction model in order to provide a visual basis for the formulation of exercise prescription. This study provides a predictive model related to necroptosis that can be useful for clinical practitioners to assess the risk of patients suffering from AD. Further studies on the regulatory function of miR-215-5p in exosomes by *CEBPB* and *GATA6* are still needed. Based on these key predictors, we revealed miRNAs that may regulate necroptosis and whose expression may be upregulated after exercise. Analysis of the transcriptional profile of skeletal muscle scRNAs helps to understand the potential mechanisms and regulatory networks underlying the upregulation of miRNAs in exosomes after exercise. Based on these mechanisms, more in-depth studies can be conducted in the future to identify specific mechanisms of exercise for AD prevention, which can provide prospective input for the development of new bionic drugs.

## Conclusion

In conclusion, exercise may help avoid necroptosis. The purpose of this study was to develop a novel AD-risk prediction model having high predictive accuracy. Exercise-induced upregulation of circulating blood exosome miR-215 expression may underlie its preventative effect on AD. miR-215 expression increases by negative feedback following a decrease in the load.

## Data Availability Statement

The original contributions presented in the study are included in the article/Supplementary Material, further inquiries can be directed to the corresponding authors.

## Author Contributions

YC contributed to the methodology, conceptualization, software, validation, formal analysis, data curation, and writing (original draft). YS contributed to the conceptualization, methodology, and supervision. ZL contributed to the methodology and conceptualization. JL contributed to the data curation and methodology. BQ and XK contributed to the data curation and writing (review and editing). CY and CG contributed to the software and validation. MY contributed to the formal analysis and data curation. XC and YW contributed to the supervision and project administration. QW contributed to the writing (original draft), conceptualization, supervision, methodology, and funding acquisition. JW and SC contributed to the conceptualization, supervision, project administration, and funding acquisition. All authors contributed to the article and approved the submitted version.

## Conflict of Interest

The authors declare that the research was conducted in the absence of any commercial or financial relationships that could be construed as a potential conflict of interest. The reviewer HX declared a shared parent affiliation with the authors YC, YS, ZL, JL, BQ, and SC to the handling editor at the time of review.

## Publisher’s Note

All claims expressed in this article are solely those of the authors and do not necessarily represent those of their affiliated organizations, or those of the publisher, the editors and the reviewers. Any product that may be evaluated in this article, or claim that may be made by its manufacturer, is not guaranteed or endorsed by the publisher.

## Footnotes

1
https://www.ncbi.nlm.nih.gov/pubmed/

2
http://www.gsea-msigdb.org/gsea/index.jsp

3
http://mirwalk.umm.uni-heidelberg.de/

4
https://www.string-db.org/

## Supplementary Material

Supplementary Figure 1 PPI network of mRNAs in the analysis of necroptosis-related lncRNAs–mRNAs correlation.

Supplementary Figure 2 Differential analysis of neuronal cell sensitivity to drugs in high and low AD risk.
