# Supplementary material for: Potential Mechanism Underlying Exercise Upregulated Circulating Blood Exosome miR-215-5p to Prevent Necroptosis of Neuronal Cells and a Model for Early Diagnosis of Alzheimer’s Disease
Source: Front Aging Neurosci. 2022 May 9;14:860364. doi: 10.3389/fnagi.2022.860364 (PMC9126031; doi:10.3389/fnagi.2022.860364)
Supplement: Supplementary file 1 [file Table_1.doc]

| **Supplementary table 1. Differential expression of miRNAs in peripheral blood of sedentary and exercise groups** | | | | |
| --- | --- | --- | --- | --- |
| miRNA | Sedentary group | Sports group | logFC | p value |
| hsa-miR-1306-3p | 2.907 | 4.321 | 0.572 | 0.020 |
| hsa-miR-215-5p | 0.517 | 2.429 | 2.232 | 0.025 |
| hsa-miR-432-5p | 8.076 | 9.207 | 0.189 | 0.032 |
| hsa-miR-129-5p | 14.659 | 15.532 | 0.083 | 0.032 |
| hsa-miR-370-3p | 3.743 | 4.882 | 0.383 | 0.047 |
| hsa-miR-197-3p | 0.317 | 1.551 | 2.291 | 0.049 |
