# Supplementary material for: Potential Mechanism Underlying Exercise Upregulated Circulating Blood Exosome miR-215-5p to Prevent Necroptosis of Neuronal Cells and a Model for Early Diagnosis of Alzheimer’s Disease
Source: Front Aging Neurosci. 2022 May 9;14:860364. doi: 10.3389/fnagi.2022.860364 (PMC9126031; doi:10.3389/fnagi.2022.860364)
Supplement: Supplementary file 2 [file Table_2.doc]

| **Supplementary table 2. Predicted results of IDH1, SIRT1 and BCL2L11 as potential target genes of hsa-miR-215-5p in the miRwalk database.** | | | | | | | | | | | |
| --- | --- | --- | --- | --- | --- | --- | --- | --- | --- | --- | --- |
| Refseqid | Symbol | Start | End | Bindingp | Energy | Accessibility | Number of pairings | Binding region length | Longest consecutive pairings | Position | Validated |
| NM_001282386 | IDH1 | 585 | 603 | 0.846 | -17.2 | 0.000 | 16 | 18 | 14 | CDS | NO |
| NM_005896 | IDH1 | 602 | 620 | 0.846 | -17.2 | 0.000 | 16 | 18 | 14 | CDS | NO |
| NM_001314049 | SIRT1 | 309 | 336 | 0.846 | -19.4 | 0.008 | 19 | 27 | 8 | CDS | YES |
| NM_012238 | SIRT1 | 1039 | 1066 | 0.846 | -19.4 | 0.008 | 19 | 27 | 8 | CDS | YES |
| NM_001142498 | SIRT1 | 520 | 547 | 0.846 | -19.4 | 0.008 | 19 | 27 | 8 | CDS | YES |
| NM_138621 | BCL2L11 | 3490 | 3551 | 1.000 | -19.1 | 0.012 | 14 | 16 | 14 | 3UTR | YES |
| NM_138622 | BCL2L11 | 3615 | 3676 | 1.000 | -19.1 | 0.012 | 14 | 16 | 14 | 3UTR | YES |
| NM_138623 | BCL2L11 | 3435 | 3496 | 1.000 | -19.1 | 0.012 | 14 | 16 | 14 | 3UTR | YES |
| NM_138624 | BCL2L11 | 3523 | 3584 | 1.000 | -19.1 | 0.012 | 14 | 16 | 14 | 3UTR | YES |
| NM_138625 | BCL2L11 | 3336 | 3397 | 1.000 | -19.1 | 0.012 | 14 | 16 | 14 | 3UTR | YES |
| NM_138626 | BCL2L11 | 3386 | 3447 | 1.000 | -19.1 | 0.012 | 14 | 16 | 14 | 3UTR | YES |
| NM_006538 | BCL2L11 | 3310 | 3371 | 1.000 | -19.1 | 0.012 | 14 | 16 | 14 | 3UTR | YES |
| NM_001204107 | BCL2L11 | 3417 | 3478 | 1.000 | -19.1 | 0.012 | 14 | 16 | 14 | 3UTR | YES |
| NM_001204108 | BCL2L11 | 3622 | 3683 | 1.000 | -19.1 | 0.012 | 14 | 16 | 14 | 3UTR | YES |
| NM_001204109 | BCL2L11 | 3511 | 3572 | 1.000 | -19.1 | 0.012 | 14 | 16 | 14 | 3UTR | YES |
| NM_001204110 | BCL2L11 | 3352 | 3413 | 1.000 | -19.1 | 0.012 | 14 | 16 | 14 | 3UTR | YES |
| NM_001204112 | BCL2L11 | 3331 | 3392 | 1.000 | -19.1 | 0.012 | 14 | 16 | 14 | 3UTR | YES |
