# Supplementary material for: Potential Mechanism Underlying Exercise Upregulated Circulating Blood Exosome miR-215-5p to Prevent Necroptosis of Neuronal Cells and a Model for Early Diagnosis of Alzheimer’s Disease
Source: Front Aging Neurosci. 2022 May 9;14:860364. doi: 10.3389/fnagi.2022.860364 (PMC9126031; doi:10.3389/fnagi.2022.860364)
Supplement: Supplementary file 3 [file Table_3.doc]

| Supplementary table 3. Worksheet of public datasets | | |
| --- | --- | --- |
| Datasets | Summary | DATABASE LINK |
| GSE33000 | An AD dataset | <https://www.ncbi.nlm.nih.gov/geo/query/acc.cgi?acc=GSE33000> |
| GSE44770 | An AD dataset | <https://www.ncbi.nlm.nih.gov/geo/query/acc.cgi?acc=GSE44770> |
| GSE144627 | A dataset of altered microRNA expression in circulating blood after exercise | <https://www.ncbi.nlm.nih.gov/geo/query/acc.cgi?acc=GSE144627> |
| GSE155933 | A skeletal muscle load-related dataset | <https://www.ncbi.nlm.nih.gov/geo/query/acc.cgi?acc=GSE155933> |
| GSE138826 | A skeletal muscle single-cell transcriptome dataset | <https://www.ncbi.nlm.nih.gov/geo/query/acc.cgi?acc=GSE138826> |
