# Supplementary figures and images for: Potential Mechanism Underlying Exercise Upregulated Circulating Blood Exosome miR-215-5p to Prevent Necroptosis of Neuronal Cells and a Model for Early Diagnosis of Alzheimer’s Disease
Source: Front Aging Neurosci. 2022 May 9;14:860364. doi: 10.3389/fnagi.2022.860364 (PMC9126031; doi:10.3389/fnagi.2022.860364)

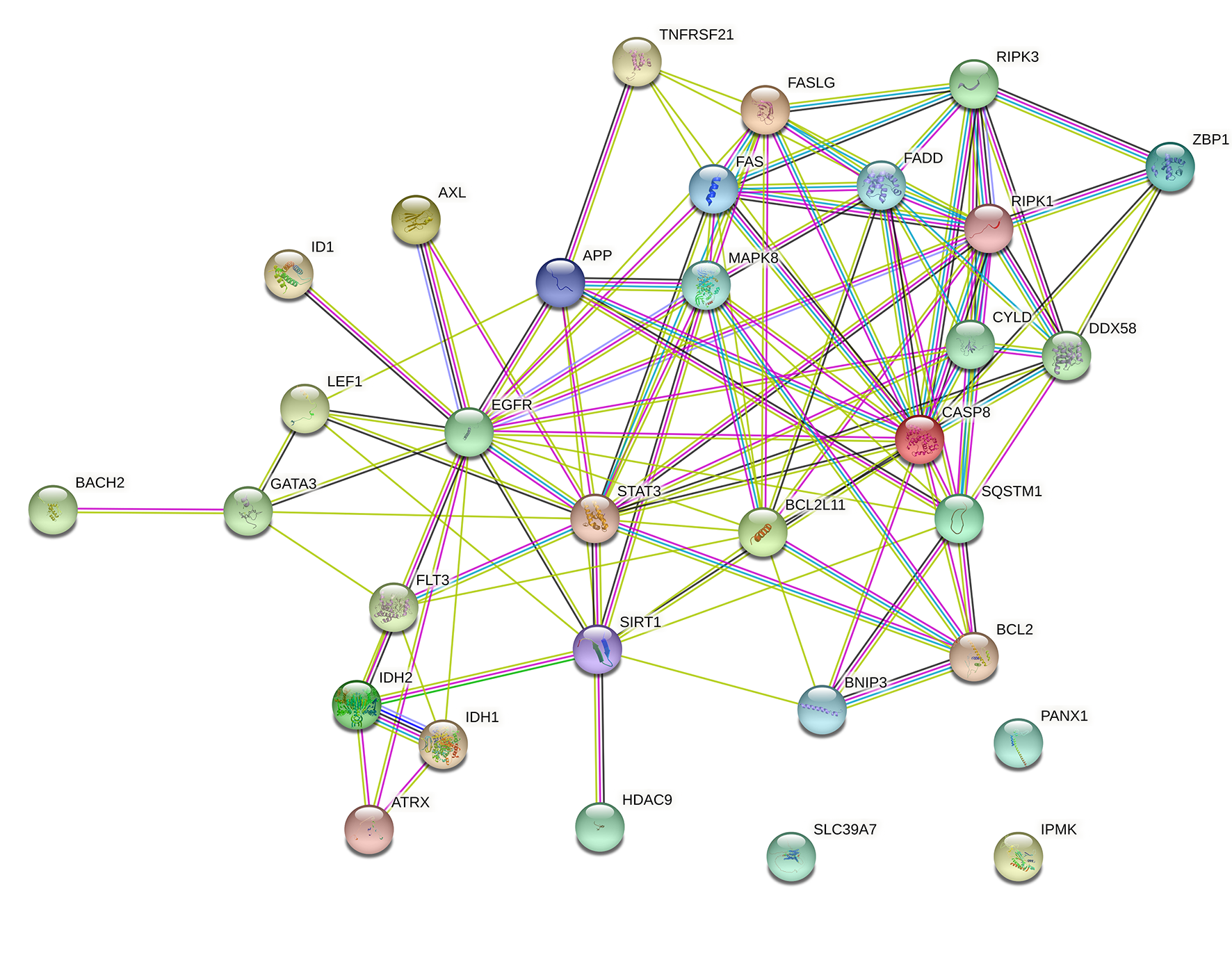

Supplement: Supplementary Figure 1 — PPI network of mRNAs in the analysis of necroptosis-related lncRNAs–mRNAs correlation. [file Image_1.TIF]

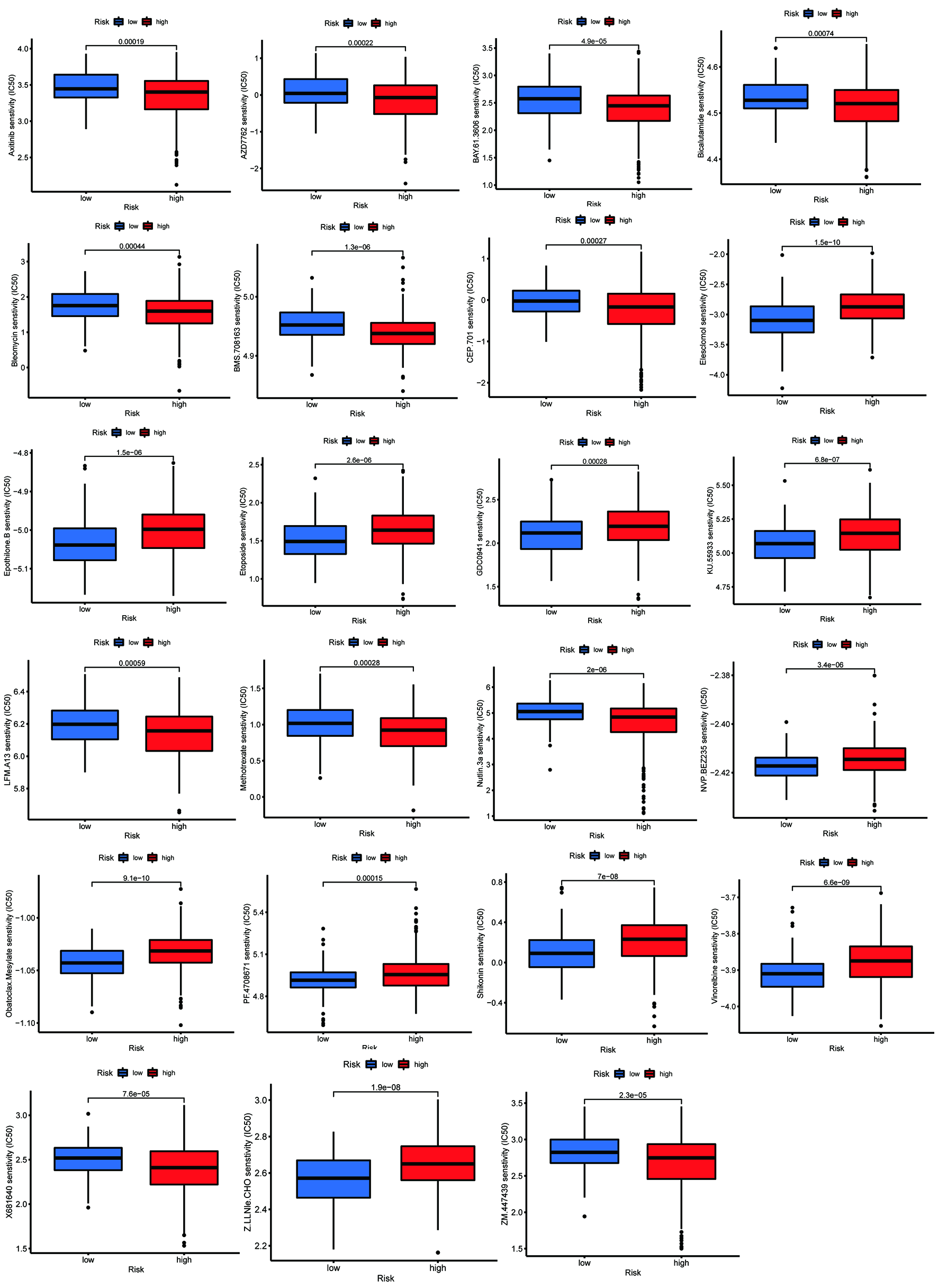

Supplement: Supplementary Figure 2 — Differential analysis of neuronal cell sensitivity to drugs in high and low AD risk. [file Image_2.TIF]
